# The Book World of Medicine and Science

**Published:** 1895-12-14

**Authors:** 


### The Book World of Medicine and Science.

Manual of Physiology. By G. N. Stewart, M.A., M.D., London. University Series. (Baillière, Tindall, and Cox. 1895.)

Publishers and authors must possess peculiar confidence in the medical student, and his capacities for absorbing physiological literature, else they would, in the face of a stationary or diminishing roll of possible purchasers, exercise some moderation in the publication of this class of text-book. Each year the budding Æsculapius meets with new difficulties in making his final selection of a suitable book to guide his studies, unless indeed—and this is often the case—he seeks the advice, and abides by the decision of his teacher. This power for good or evil vested in the hands of lecturers or professors at the large academic centres has in many cases led to the diversion of this marketable influence into immediately advantageous channels; in such cases a new text-book makes its appearance with an assured circulation at home, and a possible overflow to extraneous schools. Monographs of physiological research are one thing, and substantial physiological discoveries are another; hundreds of periodicals are annually devoted to the propagation of the former, while the results of the latter might easily be included in a few pages. Hence neither the requirements of the case nor the student's thirst for new matter can be urged in excuse for the rapid succession of books on general physiology. And for this reason authors are often hard put to it in their prefatory apologies to find adequate justification for the appearance of their views in print. In this volume the student will find all the subject matter that is necessary for his salvation at the college's examination, and in addition, will discover at the end of each section laboratory notes to assist him through a practical course explanatory and illustrative of the matter he has just read. This interpolation of practical notes is that which distinguishes Dr. Stewart's Manual from its numerous brethren. This new departure in the general arrangement of the various sections needs no apology, as the chain of argument is consecutive throughout, and logically sound. A successful attempt has been made, after the manner of Huxley or Foster, to rivet the student's attention to his subject by the adoption of an interesting and lively style, and by illustrating technical points with familar and easily understood examples. The illustrations and plates are for the most part taken from other works. Such as are original aim rather at truthful representation than schematic or diagrammatic lucidity. With a good knowledge of this manual in his head the student may face his examiner with confidence, and enter on his professional career with all the necessary physiological equipment.

Essays in Heart and Lung Disease. By Arthur Foxwell, M.A., M.D. Cantab. (London: Charles Griffin and Co. 12s. 6d.)

This book consists of a series of lectures on the subjects of heart and lung disease delivered by the author before various medical societies. If the sections on cardiac disease had been excluded, in our opinion Dr. Foxwell would have exercised a becoming modesty and a wise discrimination. Other parts of his work are so excellent that they throw a lurid light on the deficiencies of the few sections referred to. Had the above expurgation been carried out, a title referring alone to pulmonic disease might have been selected, and the contents more accurately represented by the outside description. The main subject treated of in the cardiac section is that of "high tension," but we doubt very much whether the author's views are consistent with the general acceptation of this term. For instance, he classifies the three causes of this condition in the following manner: (1) Cardiac (aortic regurgitation); (2) degeneration (atheroma); (3) vaso-motor (renal disease). The expression "tension," when applied to the circulation, designates the mean tension of the arterial contents, and not the maximum tension of the pulse wave. The pulse of aortic regurgitation is classified by Broadbent as a pulse of low tension, and the pulse of a degenerated vascular system may be either a high or low tension. The pulse of renal disease is the only one of those included in this classification which conforms to the generally-accepted meaning of the term. Some of the other lectures are well worth reading. That on the treatment of phthisis, for instance, insists strongly on the value of shaping the treatment according to the character of the sputum. The method is highly rational, but one that might degenerate into a rule of thumb practice in careless hands. The student, however, may note, with profit to himself, that the presence or absence of tubercular bacilli is not the only important factor to be noticed in sputum. As for acute pneumonia in children, although at first inclining to a belief in several distinct varieties, Dr. Foxwell now dogmatises in no uncertain terms: "There is a constitutional physical state which I propose to call catarrhal fever, which has respiratory, cutaneous, intestinal, meningeal, and renal varieties, just as there are pulmonary, anginous, nephritic, rheumatic, pyæmic, and cutaneous varieties of the constitutional pyrexial state called scarlet fever." This view we regard as a highly philosophical generalisation, and we entirely endorse his opinion. The antiseptic treatment of tubercular phthisis and the treatment of acute laryngitis will well repay the reading; and with the exception of the sections which we have, perhaps, hyper-criticised, we regard the work as an excellent addition to the literature of pulmonary disease.

